# Foreign

**Published:** 1841-02-13

**Authors:** 


### FOREIGN.

*Remarks on Stammering, by a Sufferer.*—The following observations on this annoying infirmity are from the pen of a physician, who, till his twentieth year, had been afflicted with stammering, and who then cured himself in the manner which we shall afterwards notice.

Most writers on stammering have attributed it to the irregular and convulsive movements, or to the faulty position in relation to the teeth and palate, of the tongue and lips. Thus M. Hervez de Chegoin mentions the absolute or relative smallness of the flesh of the tongue, and the shortness of the frænum as its most frequent causes; and he has reported two cases where a cure was effected by dividing this bridle.

But in a large majority of stammerers there is no irregularity whatsoever in the organs of speech: and even if there was, the admission of such a cause would not account for the infinite varieties of the infirmity and the influence which moral emotions exert upon its development.

MM. Sauvage and Itard regard debility of the muscles of the tongue as one of the most frequent causes. But do not persons that stammer execute with the greatest ease and rapidity all the various movements of the tongue and lips?

Is it not rather a state of spasm than of debility? How, too, on this hypothesis, are we to explain the spontaneous cure of stammering as life advances, when debility, local as well as general, must necessarily increase?

Other writers seek for the cause of the malady in the action of the brain on the muscles of the voice—an explanation which may be quite just, indeed, if it were not so vaguely expressed: a vagueness which seems to have actually bewildered some writers who have adopted the explanation in question. Thus, Rullier, in the Dictionnaire de Medecine, says: "In stammering, the cerebral irradiation which follows an act of thought, and becomes the moving principle to set in action the muscles necessary to the oral expression of the ideas, bursts forth with such impetuosity, and is reproduced with such rapidity, that it exceeds the mobility of the agents of articulation."

But this is surely paying too great a compliment to stammerers; for my part I must confess that I never experienced that rapid flow of ideas and impetuous action of brain, which the author talks of.

M. Magendie attributes stammering to such a deficient action of the organic intelligence as is capable of disturbing the movements of the vocal organs, which are influenced at the same time by the nervous system of organic life, and by the action of the brain.

All that we can reply to this explanation is, to ask what the author means to announce; for to us his language is quite unintelligible.

But to form a more accurate idea as to the cause of stammering, let us now briefly notice what we observe in a person afflicted with this infirmity when he attempts to speak.

In some cases—and this is the least severe form of the malady—the person speaks sufficiently fluently for a greater or less length of time; but if the pronunciation of a word which begins with certain consonants, such as *b*, *p*, or *v*, coincides with the close of an expiration, then the breathing becomes embarrassed, hurried, and panting, and the efforts of the person to pronounce the word are accompanied with convulsive movements of the lips; at other times the lips are in a state of tonic spasm. At length the person succeeds in overcoming this difficulty, by taking his breath or making an inspiration.

In other cases, where the stammering is more decided, the person often cannot utter without difficulty almost any sound, even when the word which he wishes to pronounce begins with a vowel, (as was the case with myself in respect to the word *oüi*;) the features and neck swell up; the jugular veins become distended; and the person tosses his arms about to find relief.

These two degrees of the infirmity are often observed to co-exist in the same person: if he becomes embarrassed by a word which begins with a consonant which is not easily pronounced, the fear of not being able to proceed renders the respiration panting; and then any efforts at speech are quite impracticable.

In both degrees or stages of the malady, *the stammering ceases after a strong inspiration;* but it returns at once if the person does not continue to respire regularly, and the more frequently and badly if his breathing becomes agitated, and he strives much against it.

*The great object, therefore, is to bring and keep the respiration in a quiet, tranquil condition, and cause it to follow a certain degree of cadence, such as we use in singing or in declamation. If the person can but once effect this, he will no longer stammer.*

From what we have now said, it is evident that in all the various sorts or degrees of stammering, its essential cause consists in a convulsive condition, either of a tetanic or a choreic character, of the respiratory function—the effect of which is, either to disturb more or less, or altogether prevent, the articulation of sounds.

What occurs in the lips, tongue, and throat, is not the essential and primary cause, but is altogether accessory; and it is according to which of these parts is chiefly affected, that the different varieties of the infirmity are produced.

The immediate cessation of the irregular convulsive movements by taking in a deep inspiration, clearly shows how intimately they are connected with the cause now mentioned. It was by attending to this simple means, suggested to me by Dr. Lindt, of Berne, that I cured myself at the age of twenty years. The cure, indeed, was aided a good deal, I believe, by the employment at the same time of gymnastic exercises—the effect of which is powerfully to increase the influence of the will over the muscular system in general. Indeed it has been by their influence in regulating the act of breathing, that the efficacy of various means, which have been recommended and found useful, at various times, in the treatment of stammering, is to be accounted for. In this way the practice of declaiming on the sea-shore with a pebble in his mouth cured, it is well known, Demosthenes of old.

M. Itard has advised the use of some mechanical means to keep the tongue raised, and he makes his patients at the same time speak in a foreign language. This plan of keeping the tongue raised towards the palate has been recommended by other writers. By this simple means, Madame Leigh assures us that she effected a cure in upwards of one hundred and fifty cases.

But however useful it may be to a person who stammers to acquire a certain control over the movements of the tongue, let it be ever kept in mind, that no permanent benefit will be derived unless the patient acquires, at the same time, the power of regulating his breathing—so that whenever he begins to hesitate or falter, he at once draws in a full breath. Madame Leigh imposed upon her pupils absolute silence, except during the hour of practising: and she always observed that those who had the greatest mental energy and power over their will were the most easily and quickly cured.

M. Columbat, although his theory of stammering is far from being correct in our opinion, has recommended a most judicious method of treatment, which if rightly pursued is always sure to effect a cure. It consists in adopting the following three means: 1, giving the tongue such a position that its apex is directed upwards and backwards; 2, taking in a deep breath at the commencement of each phrase, and repeating this more or less frequently; and 3, marking the time in speaking by the movements of the thumb upon the fore-finger.

By following out these simple rules, it is almost impossible for any one to stammer; and when we consider that this method of treatment consists essentially in causing the person to inspire at the commencement of each phrase, and marking the regular return of the inspirations by the movements of the thumb upon the finger, we at once understand its physiological explanation.

M. Serres' plan consists in making the person accompany the utterance of each sound with a powerful movement of the arms; he does not trouble himself about the situation of the tongue. Now this plan will often succeed as well as that which we have just recommended.

But neither M. Serres, nor M. Itard, his reporter, who is surprised at the singularity of this method, seem to have perceived that every muscular effort is in fact made during the act of expiration, and therefore that the pupil, to enable him to accompany the emission of any sound with a movement of his arms, must have always a provision of air in his lungs, and that thus he acquires the custom of speaking only during expiration.

There cannot be a question as to the utility of gymnastic exercises in increasing the energy of the will and the action of the brain upon the whole muscular system. It is a novel and a most practically useful idea of M. Serres, that the treatment of stammering should consist in a *gymnastique* of the respiratory and the vocal organs.—*Med. Chir. Rev.*, *from Gazette Medicale.*

---

*On Artificial Nipples of Ivory.*—The following remarks are from a report of Messrs. Dubois, Capuron and Villeneuve—three of the most distinguished accoucheurs of Paris—on the artificial nipples recently invented by M. Charriere, an ingenious cutler of the metropolis.

"In the construction of these nipples, he uses ivory which has already been made soft and flexible by a process that has been long known: they are more solid and more durable than those hitherto employed. They are sufficiently resisting not to be flattened by the lips of the infant, and yet not too hard to fret and inconvenience them. They are easily kept clean by merely shaking them about in water, and with this simple precaution it will be found that they are not apt to communicate any unpleasant smell or taste to the milk. Besides, they can be readily attached to any sort of sucking dish or bottle, if the child is brought up by the hand and not suckled by the mother.

To preserve the flexibility of the prepared ivory, all that is necessary is to keep it from the contact of the air by placing the nipple under a glass, or by wrapping it round with a damp cloth. In fact we (the reporters) think highly of these new contrivances, and already, we are told, they are extensively used both in public establishments and in private practice."

(We have observed in some of the recent French journals, that the prepared flexible-made ivory has been manufactured into bougies for the male uretha; but whether they possess any peculiar advantages we do not know.

Excellent artificial nipples are now made with prepared caoutchouc: they are entirely destitute of any unpleasant smell or taste, and have quite a fleshy feel to the lips when sucked with.—*Rev.*)

*German Treatment of Local Paralysis.*—Several German writers very strongly recommend the use, internally as well as externally, of phosphorus in cases of paralysis of the optic and auditory nerves.

In a case related by M. Dezeimeris in his late elaborate memoir on diseases and injuries of the frontal sinuses, where deafness and blindness on the left side supervened upon a severe injury of the head, he resorted to this treatment with success.

He prescribed as follows:

℞. Phosphori gr. j. solve in
Olei animalis æther. ʒj.
Olei. caryophyll. ℈j.

Dose.—Three drops, to be gradually increased to 20, to be taken on a piece of sugar night and morning. And

℞. Phosphori gr. ij.

Olei animalis æther. ʒj.

Olei cajeputi ʒss. Solve.

The eyelids to be well rubbed with this embrocation three or four times daily.

A blister also was applied upon the mastoid process and kept open.

In four weeks the patient had quite recovered his hearing and sight.—*Med. Chir. Rev. from L'Experience.*

---

*Case of Imperforate Vagina, and Menstruation (from the Bladder?)*—Ellen S——, æt. forty, and married ten years, presented herself as an out-patient at St. Thomas's Hospital, under the care of Dr. Cape, on Friday, April 24, 1840. She complained of a hard and painful tumour, about the size of a melon, situated in the uterine region, which could be felt above the pubis. She had no discharge, and her general health was not impaired. On examination per vaginam being attempted, no vagina could be found. She was then requested to retire to one of the female wards, where she submitted to an ocular examination; the labia, nymphæ, clitoris, and all the external organs of generation, were quite normal, with the exception of the vagina, which was totally imperforate, not amounting even to a cul-de-sac; being perfectly flat and unyielding, with a granulated red surface. On examining per anum, the tumour could be very distinctly felt, painful on pressure, and apparently of a subcartilaginous character. She has menstruated regularly ever since she was thirteen, the period lasting four days, and of the usual quantity.

On the 15th of May, the catamenia being present, it was thought a good opportunity for ascertaining whence the discharge flowed, as, on the previous examination, the only aperture visible was the orifice of the urethra; when it was seen distinctly exuding guttatim from the meatus urinarius.

*Medical Gazette, June* 26, 1840.

